# Kidney Dysfunction In Young People Living With HIV On Dolutegravir-Based Regimens In Kampala, Uganda

**DOI:** 10.21203/rs.3.rs-9091584/v1

**Published:** 2026-03-20

**Authors:** Esther M Nasuuna, Risa Hoffman, Robert Kalyesubula, Chido Dziva Chikwari, Helen A Weiss

**Affiliations:** Non-communicable Diseases Program, Medical Research Council/Uganda Virus Research Institute and London School of Hygiene and Tropical Medicine Uganda Research Unit; David Geffen School of Medicine, University of California Los Angeles; Non-communicable Diseases Program, Medical Research Council/Uganda Virus Research Institute and London School of Hygiene and Tropical Medicine Uganda Research Unit; Biomedical Research and Training Institute; MRC International Statistics and Epidemiology Group, London School of Hygiene & Tropical Medicine

**Keywords:** Kidney function, dolutegravir, young people living with HIV, Sub Saharan Africa

## Abstract

We assessed the association between dolutegravir (DTG)-based antiretroviral therapy and kidney abnormalities among young people living with HIV aged 10–24 years in Kampala, Uganda. In this cross-sectional study, albumin-creatinine ratio (ACR), proteinuria, and estimated glomerular filtration rate (eGFR) were measured. Among 483 participants, 78% received tenofovir (TDF)/DTG. Mean serum creatinine was higher and creatinine-based eGFR lower among those on TDF/DTG, while cystatin C and cystatin C-based eGFR were similar. The prevalence of elevated ACR, proteinuria, and eGFR <90 ml/min/1.73m^2^ was similar. Kidney abnormalities were common, supporting the need for longitudinal studies to clarify chronic kidney disease risk.

## Introduction

Antiretroviral therapy (ART) has transformed HIV infection from a fatal illness into a chronic condition [1]. The success of ART means that young people living with HIV (YPLHIV) are increasingly surviving into adulthood and face long-term health challenges [1]. Non-communicable conditions, including chronic kidney disease, have emerged as important contributors to morbidity and mortality [2]. Multiple factors-direct effects of HIV, coexisting infections, traditional risk factors (hypertension and diabetes), and exposure to nephrotoxic ART-drive kidney dysfunction in YPLHIV [3]. Young people are especially vulnerable because they are likely to be exposed to ART for many decades, during which cumulative drug effects and ongoing HIV-related immune activation may increase the risk of kidney dysfunction [4].

The recent global transition to dolutegravir (DTG)-based regimens has introduced new complexities in monitoring kidney function. DTG is now the preferred first-line ART in many low- and middle-income countries, including Uganda, because of its high efficacy and favourable tolerability profile [5]. However, DTG inhibits tubular secretion of creatinine without affecting glomerular filtration, leading to modest but persistent increases in serum creatinine [6]. This pharmacological effect may confound the assessment of kidney function and obscure early signs of true kidney impairment. There is a critical need to characterise the burden of kidney dysfunction in YPLHIV on DTG-based ART in Uganda. We set out to describe the prevalence and association of kidney dysfunction with DTG-based regimens among YPLHIV in Uganda.

## Methods

We conducted a cross-sectional study in seven urban public health facilities in Kampala, Uganda, which provide comprehensive HIV care and treatment services, mostly for the urban poor.

The study included YPLHIV aged 10–24 years with presumed perinatal HIV infection. Pregnant YPLHIV were excluded. Systematic random sampling (listing all YPLHIV in the clinics and picking out every other name) was used to identify potential participants. These were invited to join the study. Details of the parent study have been published previously [7].

Eligible participants were invited to the HIV clinic through a phone call to those above 18 years or the caregiver for those below 18 years. They were screened, consented, and enrolled. We collected urine samples for the albumin creatinine ratio (ACR), proteinuria on dipstick, and blood samples for serum creatinine and cystatin C. We estimated glomerular filtration rate (eGFR) using the CKDEPI 2021 equation or the Bedside Schwartz (< 18 years).

Participants were interviewed to collect socio-demographic data such as age, sex, and socioeconomic status. Measurements were taken for weight, height, mid-upper arm circumference (MUAC), body mass index (BMI) was calculated as weight over height squared. Stunting was calculated according to the WHO standards [8]. Muscle mass was determined using a bioimpedance machine and calculated using sex and age-specific cut-offs.

### Data management and statistical analysis

Data were collected in REDCap and analysed with STATA statistical software Version 18 (STATA Corp USA). Demographic and clinical data were summarised in percentages or means (standard deviation [SD]) and median (interquartile range [IQR]). We used chi-square tests to compare kidney function parameters of participants on tenofovir disoproxil fumarate (TDF), lamivudine (3TC), and DTG compared with those on DTG combined with other Nucleoside Reverse Transcriptase Inhibitors, such as zidovudine or abacavir, and 3TC (other DTG regimens).

### Ethical considerations

Ethical approval was received from the Uganda Virus Research Institute (UVRI) Research Ethics Committee (reference number GC/127/946), the Uganda National Council of Science and Technology (HS2578ES) and the London School of Hygiene and Tropical Medicine institutional review board (28797). All participants aged 18 years or older provided written informed consent. Those aged < 18 years, provided written participant assent, and caregiver consent. All participants with suspected CKD were referred to a nephrologist for management.

## Results

Out of 500 YPLHIV, 483 were enrolled into the study, with 367 (76%) on TDF/3TC/DTG and 116 (24%) on other regimens. There were slightly more females than males in both groups. There were more underweight participants on the non-TDF-containing DTG regimens. Viral suppression, mean duration on ART, and muscle mass were similar in both groups. Table 1

### Participants’ kidney parameters by regimen

The mean serum creatinine levels were higher among participants on TDF/DTG compared to those on other DTG-based regimens without TDF, although the mean eGFR and the proportions with eGFR < 90 ml/min/1.73m^2^ were similar. The mean cystatin C was similar in both groups. The mean eGFR was within normal limits and lower among those on TDF/DTG compared to those on other DTG-based regimens. Although the proportion with eGFRcystc below 90ml/min/1.73m^2^ was higher among those on DTG-based regimens without TDF compared to those on TDF/DTG, this was not significant. The proportion of YPLHIV with ACR>30mg/g was higher among those on other DTG-based regimens, and proteinuria proportions were high but similar (Table 2).

## Discussion

Our data suggest a high prevalence of kidney dysfunction (based on eGFR cystatin C below 90ml/min/1.73m^2^ and proteinuria) among YPLHIV compared to youth who are not living with HIV, which is not associated with DTG/TDF versus other DTG-based regimens.

Our findings are consistent with those of the ODYSSEY trial from Uganda, Thailand, Europe, Zimbabwe, and South Africa, which reported no kidney-related adverse effects in 707 children and adolescents living with HIV on DTG-based regimens coupled with NRTIs, including TDF at 96 weeks [9]. A systematic review on the efficacy and safety of DTG among children and adolescents reported data on 11 studies, which did not report any kidney-related adverse events, although the number of children on both DTG and TDF and the duration were not reported [10]. Taken together, these data are reassuring about ongoing use of DTG combined with TDF as first-line ART for YPLHIV initiating treatment in Uganda [5].

There are concerns about DTG obscuring kidney dysfunction, especially when it is co-administered with TDF, which is known to cause kidney dysfunction [11, 12]. This concern has not yet been fully addressed, as there are very few studies done in Africa that involve young people living with HIV on both DTG and TDF [13]. A study done in Nigeria among 170 adult PLHIV found that those on TDF/DTG had impaired kidney function [14]. A pilot clinical trial showed that PLHIV who switched to TDF/DTG combination had a greater decline in eGFR compared to those switched to DTG/lamivudine [15]. A global systematic review and meta-analysis showed a 7% prevalence of chronic kidney disease among those treated with TDF, there was no mention of DTG effects [16]. Since the duration on ART is associated with risk of TDF kidney toxicity [17], and YPLHIV are exposed to TDF for a longer time than adults living with HIV, vigilant monitoring of kidney function is warranted for YPLHIV treated with TDF-containing regimens, to ensure early diagnosis. Use of tenofovir alafenamide (TAF) should also be considered, as this is associated with less kidney toxicity compared to TDF, according to data pooled from 26 clinical trials that also included children [18]. However, weight and metabolic changes should be monitored [19].

The main limitation of our study was the inability to compare the kidney function of YPLHIV on DTG based regimens to others who were not exposed to DTG, since most YPLHIV were switched to DTG following the WHO recommendations. We did not account for the regimen changes over time with the changing recommendations over the lifetime of the YPLHIV. It was also a cross-sectional study, and more information about long-term toxicity should be collected from longitudinal studies. The strengths are that the study was adequately powered to detect an association, and our findings are generalisable to YPLHIV living in settings similar to ours.

## Conclusion

We found that YPLHIV on DTG-based regimens had a high prevalence of proteinuria and elevated ACR regardless of the nucleoside analogues utilized alongside DTG. However, eGFR calculated from both serum creatinine and cystatin C was largely within normal limits. Longer-term follow-up is needed to understand whether YPLHIV will develop chronic kidney disease as they age, and to elucidate risk factors for kidney disease progression, including safer long-term ART regimens.

## Figures and Tables

**Table 1:** Demographic and clinical characteristics of the participants by ART regimen

Mean Age in years (SD)	TDF/DTG N = 367	Other/DTG N = 116	p-value
	17.0 (3.5)	14.4 (3.4)	< 0.001
10 to 17	231 (62.9%)	93 (80.2%)	<0.001
18 to 24	136 (37.1%)	23 (19.8%)	
Sex			
Female	211 (57.5%)	62 (53.4%)	
Socioeconomic status			
Lowest	129 (35.1%)	44 (37.9%)	0.86
Middle	114 (31.1%)	35 (30.2%)	
Highest	124 (33.8%)	37 (31.9%)	
Body mass index			
Normal	224 (61.2%)	44 (37.9%)	<0.001
Underweight	106 (29.0%)	67 (57.8%)	
Overweight	36 (9.8%)	5 (4.3%)	
Stunting			
Not stunted	221 (84.0%)	80 (79.2%)	0.28
Stunted	42 (16.0%)	21 (20.8%)	
Mid upper arm circumference (MUAC)			
Normal	338 (92.3%)	100 (86.2%)	0.05
Malnourished	28 (7.7%)	16 (13.8%)	
Weight (kg)	51.2 (11.5)	41.9 (12.7)	<0.001

Malnourished participants had a MUAC in either yellow or red.

**Table 2:** Participant kidney parameters by ART regimen

Variable	TDF/DTG N = 367	Other DTG-based* N = 116	p-value
Serum creatinine Mean (SD)	0.68 (0.15)	0.59 (0.14)	<0.001
eGFR serum creatinine (SD)	118.8 (19.3)	113.9 (20.1)	0.32
eGFR serum creatinine			
> 90ml/min	317 (87.3)	103 (89.6)	0.52
< 90ml/min	46 (12.7)	12 (10.4)	
Cystatin C Mean(SD)	0.81 (0.13)	0.81 (0.12)	0.78
eGFR cystatin C (SD)	114.2 (15.2)	116.5 (15.3)	0.15
eGFR cystatin C			
Above 90ml/min	212 (58.4)	60 (52.2)	0.47
Below 90ml/min	151 (41.6)	55 (47.8)	
Albumin creatinine ratio			
Below 30mg/g	328 (90.4)	100 (87.0)	0.29
Above 30mg/g	35 (9.6)	15 (13.0)	
Proteinuria			
Negative	263 (71.7)	81 (69.8)	0.70
Positive	104 (28.3)	35 (30.2)	

* Regimens with either abacavir or zidovudine together with lamivudine and dolutegravir.

## Data Availability

To protect the confidentiality of the participants who provided the clinical data, the data that support the findings of this study are available from the corresponding author upon reasonable request.

## Abbreviations

ACR: Albumin Creatinine Ratio
ART: Anti-Retroviral Therapy
BMI: Body Mass Index
CKD: Chronic Kidney Disease
CKD-EPI: Chronic Kidney Disease Epidemiology Collaboration
DTG: Dolutegravir
eGFR: Estimated Glomerular Filtration Rate
HIV: Human Immunodeficiency Virus
IQR: Interquartile Range
KDIGO: Kidney Disease Improving Global Outcomes
MUAC: Mid upper arm circumference
SD: Standard Deviation
SSA: Sub-Saharan Africa
USA: United States of America
WHO: World Health Organization
YPLHIV: Young People Living with HIV
